# The anti-cancer effects of fucoidan: a review of both in vivo and in vitro investigations

**DOI:** 10.1186/s12935-020-01233-8

**Published:** 2020-05-07

**Authors:** Yuan Lin, Xingsi Qi, Hengjian Liu, Kuijin Xue, Shan Xu, Zibin Tian

**Affiliations:** The Affiliated Hospital of Qingdao University, No.16 Jiangsu Road, Shinan Disrtict, Qingdao, China

**Keywords:** Fucoidan, Bioactivity, Anticancer, Apoptosis, Cell cycle arrest, Adjuant

## Abstract

Fucoidan is a kind of the polysaccharide, which comes from brown algae and comprises of sulfated fucose residues. It has shown a large range of biological activities in basic researches, including many elements like anti-inflammatory, anti-cancer, anti-viral, anti-oxidation, anticoagulant, antithrombotic, anti-angiogenic and anti-Helicobacter pylori, etc. Cancer is a multifactorial disease of multiple causes. Most of the current chemotherapy drugs for cancer therapy are projected to eliminate the ordinary deregulation mechanisms in cancer cells. Plenty of wholesome tissues, however, are also influenced by these chemical cytotoxic effects. Existing researches have demonstrated that fucoidan can directly exert the anti-cancer actions through cell cycle arrest, induction of apoptosis, etc., and can also indirectly kill cancer cells by activating natural killer cells, macrophages, etc. Fucoidan is used as a new anti-tumor drug or as an adjuvant in combination with an anti-tumor drug because of its high biological activity, wide source, low resistance to drug resistance and low side effects. This paper reviews the mechanism by which fucoidan can eliminate tumor cells, delay tumor growth and synergize with anticancer chemotherapy drugs in vitro, in vivo and in clinical trials.

## Background

Cancer is a multifactorial disease of multiple causes. It is mainly caused by acquired genetic changes, resulting in tumor cells gaining survival or growth advantages [1]. Its occurrence is a complicated process with multiple factors and steps, which is closely related to infection, smoking, occupational exposure, environmental pollution, unreasonable diet, genetics and other factors [2–4]. It has biological characteristics such as cell differentiation and proliferation abnormality, loss of growth control, invasiveness and metastasis [5]. Tumor metastasis is one of the important causes of cancer patients’ death [6]. Abnormal intracellular signal transduction and continuous activation of cellular pathways are usually closely related to tumor cell proliferation and survival. For example, the PI3K-AKT-mTOR signaling pathway has attracted much attention due to its involvement in the regulation of various cellular functions including messenger RNA(mRNA) translation, cell cycle regulation, gene transcription, apoptosis, autophagy and metabolism [7]. At present, the treatment of cancer mainly depends on surgery, radiotherapy and chemotherapy. But the side effects are serious, so the curative effect is limited. Therefore, the search for low toxicity natural substances is one of the current research priorities of scientists. It has been found that some natural extracts targeted specific signaling pathways can inhibit or delay the carcinogenesis process at different stages and have the characteristics,such as targeting specificity, low cytotoxicity, and easy induction of cancer cell apoptosis [8].

Fucoidan has been used as a medicinal nutritional supplement in Asia for a long time due to its medicinal characteristics, including anti-cancer action. It is a category of sulfated carbohydrates that are derived from marine brown algae [9]. The anticancer activity of fucoidan has been widely researched and the earliest research reports appeared in the 1980s [10]. A large number of experiments show that fucoidan may go against the tumor cells proliferation and the growth or metastasis of tumors by inducing cell apoptosis and inhibiting angiogenesis [11]. This review summarizes fucoidan’s anti-cancer therapeutic potential as a natural marine drug based on recent advances from in vitro and in vivo experiments.

## Fucoidan

### Sources and structure

Brown algae, seaweeds that are widely distributed in various cold sea areas, are a large group of marine plants, mainly including Sargassum, Fucus, etc. Brown algae are also rich in active substances, such as polysaccharides, terpenoids, proteins, polyphenols, sterols, the multi ring sulfurous sulfid cyclics, macrolides, trace elements and fucoidan is one of them [12]. Fucoidan is a stick–slip component that derived from the surface of brown algae. People generally use water, dilute acid or alkali to extract fucoidan from seaweeds, but these methods usually take a long time and large amounts of reagents [13]. With the continuous progress of science and technology, people have improved the traditional extraction methods and developed some new methods. Microwave or ultrasound is used to drive the water molecules in cells to vibrate, thereby breaking the cells and improving the efficiency of traditional water extraction method [14]. Enzyme-assistant extraction is to use enzyme to dissolve the cell wall and release the cell contents. This method has high catalytic efficiency and specificity [15].

**Fig. 1:**
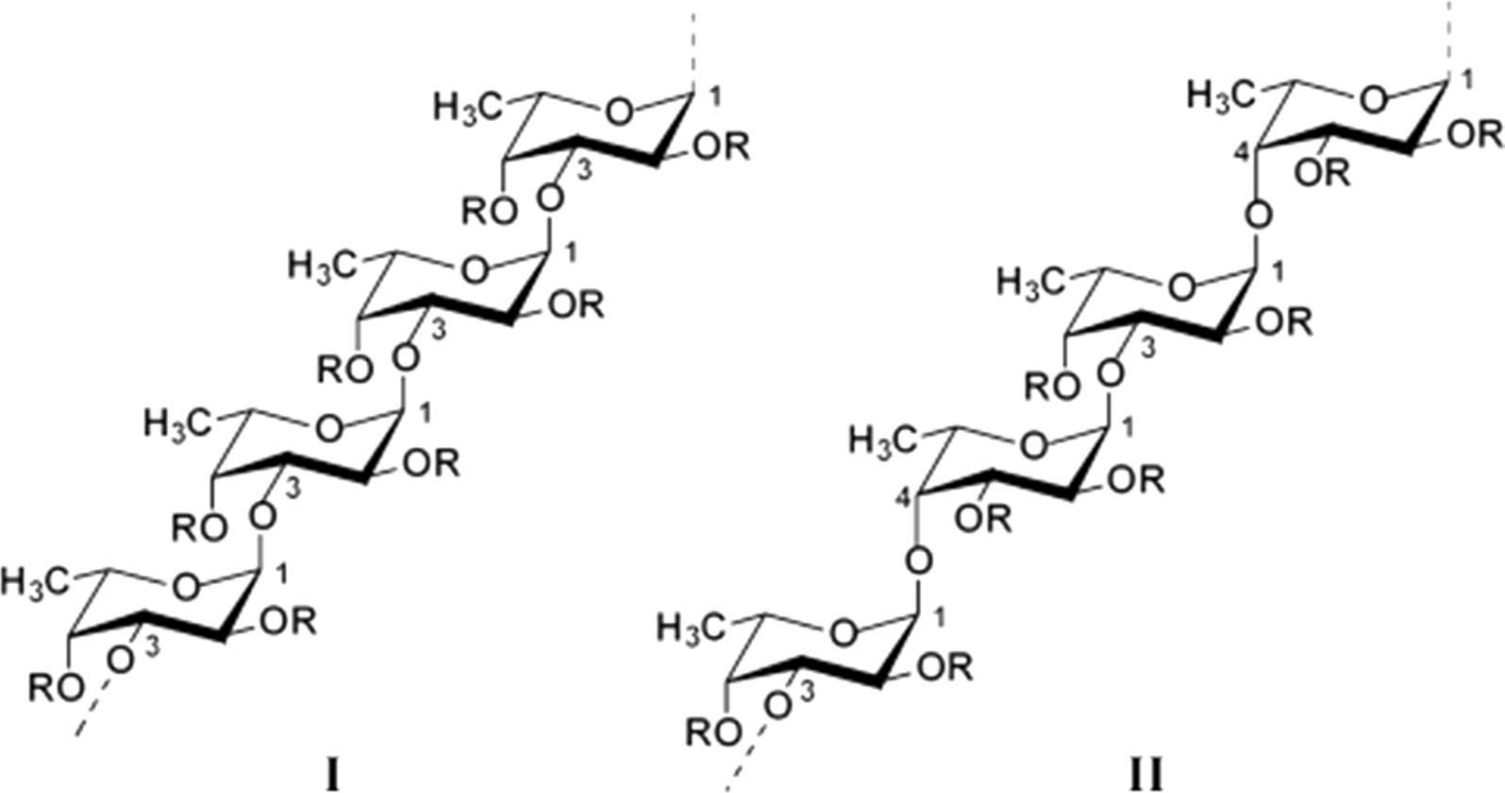
2 sorts of homofucose backbone chains of fucoidan [16]. R [II] describes the potential attnachment sites of carbohydrate (α–l-fucopyranose, α–d-glucuronic acid) and non carbohydrate (sulfate and acetyl) substituents [16]

The fuoidan’s chemical structure is complicated, which contained two major backbons, chains (I) is only formed by (1 → 3)-linked α-l-fucopyranose residues. However, chains (II) consists alternately of (1 → 3) or (1 → 4)-linked α-l-fucopyranose residues (as shown in Fig. 1) [16]. The content of α-l-fucose in fucoidan is 34–44%. Likewise, it consists of other monosaccharides including galactose, xylose, mannose, uronic acid, etc. All of them, however, account for below 10% of the whole polysaccharide formation [17]. The sulfuric acid group is mostly located at the C-4 stance, while only a few are located at the C-3 position [18, 19]. It is one kind of natural heteropolysaccharide [20, 21].

### Dose and route of administration

Because of the different source and purification methods, the dosage of fucoidan varied greatly in vitro experiments. Hsu et al. treated A549 lung cancer cells with fucoidan then they found that fucoidan inhibits 50% of cell proliferation of A549 after 48 h (the concentration is only 100 μg/mL) [22]. While in another research, Wilfred et al. discovered that fucoidan at the dose of 700 μg/mL can inhibit 50% of cell proliferation of the same cancer cells after 48 h [23]. Different sources of fucoidan may be the main cause of the difference.

The *in* *vivo* experiments in mice showed that the source, dosage, frequency of administration and route of administration of fucoidan may lead to different antitumor activity. Fucoidan’s antitumor activity was studied by Alekseyenko et al. in C57 mice transplanted with Lewis lung adenocarcinoma. The results showed that a single injection of 25 mg/Kg of fucoidan possessed no substantial inhibitory impact on tumour increment, while the mice were well tolerated with repeatedly injecting fucoidan using a dose of 10 mg/kg, and the drug showed significant anti-tumor (the tumor growth inhibition rate was 33%) and anti-metastatic activity (29% reduction) [24]. Most in vivo experiments have been administered by intraperitoneal injection, and the addition of fucoidan in food, gavage, subcutaneous injection, intravenous injection, etc. have also been deeply studied. Current researches indicated that different routes of administration make the concentration and metabolic rate of fucoidan in the body significantly different, which in turn has different effects on the occurrence and development of tumors [25–27].

### Metabolism and toxicity

In the past few decades, it was generally believed that large-molecular-weight fucoidan could not be absorbed by human intestine due to the lack of the corresponding digestive enzymes. As a result, the mechanism of antitumor effect of fucoidan by oral administration is still unclear [28]. In 2005, the clinical study of the fucoidan’s absorption through the human gut was firstly reflected by Irhimeh et al. [29]. Kizuku et al. used the fucoidan-specific antibodies extracted from *Cladosiphon okamuranus* (Okinawa Mozuku) in their laboratory with the sandwich Elisa method for fucoidan research to examine the absorption of this particular source’s fucoidan in intestine of rats. Their results illustrated that the fucoidan could be absorbed by intestinal macrophages and Kupffer cells [30, 31]. In a clinical trial involving 396 Japanese volunteers, which is designed and completed by the same research group, fucoidan was detected in 385 people’s urine after fucoidan’s oral administration, and the concentration was significantly different. The concentration of fucoidan in urine is mainly related to whether they live in Okinawa prefecture. The volunteers living in Okinawa region have the habit of eating Mozuku [32]. In 2010, Hehemenn et al. found that seaweed digestive enzymes were detected in Japanese people who frequently consumed seaweed, however, those enzymes were rarely found in North Americans who did not prefer seaweed [33]. This also explains why volunteers living in the Okinawa region have higher absorption of fucoidan. After oral administration of fucoidan, the enzymes present in the intestine will help to absorb the fucoidan, which accumulates in the liver and slowly excretes with the urine [32].

Most in vitro experiments have demonstrated that fucoidan with the cytotoxic concentration on tumour cell lines has no effect on normal cell growth and mitosis [34, 35]. In an in vivo experiment in Wister rats, 300 mg/kg was administered by oral gavage daily for 6 months and no significant adverse effects were found. Nevertheless, when the researchers increased the dose to 900–2500 mg/kg, it caused coagulopathy and the clotting time was significantly prolonged [36]. In another in vivo experiment in Sprague–Dawley rats, researchers didn’t observe significant side effects when taking 0–1000 mg/kg fucoidan orally for 28 days. Then they increased the concentration to 2000 mg/kg, plasma ALT was significantly elevated [37]. In a trial of the combination of fucoidan and cyclophosphamide, injecting fucoidan with 25 mg/kg only once did not prevent tumor growth of mice, and 3 of 10 mice died. When cyclophosphamide was administered in combination, 7 of 10 mice died and no mice died when cyclophosphamide was used alone [24]. In Naoki et al. study, the participants ingested 5 capsules contained 166 mg of fucoidan daily for up to 12 months. No obvious adverse reactions were detected in all participants [38]. In a similar experiment by Natsumi et al., the subjects took 6 g fucoidan a day for 6–13 months, and no significant adverse reactions were observed [39]. The results suggest that daily oral administration of a certain dose of fucoidan for 1 year is safe and tolerable.

### Therapeutic effects

The anticancer activity of fucoidan has been extensively studied, and the earliest research report have appeared in the 1980s. Since then, a huge quantity of studies have revealed that fucoidan can directly exert anti-cancer effects through cell cycle arrest, induction of apoptosis, etc., and can also indirectly kill cancer cell by activating natural killer cells, macrophages, etc. [40, 41]. In addition, fucoidan possesses a good many biological activities, such as anti-inflammatory, anti-oxidation, anti-clotting, anti-thrombosis, anti-viral, anti-angiogenesis, anti-Helicobacter pylori and so on [19, 42–44]. Compared with chemically synthesized drugs, natural extracts are used as novel antitumor drugs or as adjuvants in combination with antitumor drugs because of their high biological activity, wide range of sources, low drug resistance and low side effects. Fucoidan had shown antioxidant activity in some research. It can scavenge excess free radicals and is an excellent natural antioxidant. The low molecular weight fucoidan were separated into DF1, DF2 and DF3 after processing. They all possessed certain superoxide anion radical scavenging activity [45]. It had been found that the anti-viral activity of fucoidan is closely related to its sulfate content. The higher mass fraction of sulfate groups, stronger the anti-viral activity [46]. However, the molecular weight and structure of fucoidan obtained by different extraction methods are different, and they will have certain effects on their biological activities [47, 48].

## Fucoidan and cancer

### The anticarcinogenic mechanism of fucoidan

**Fig. 2:**
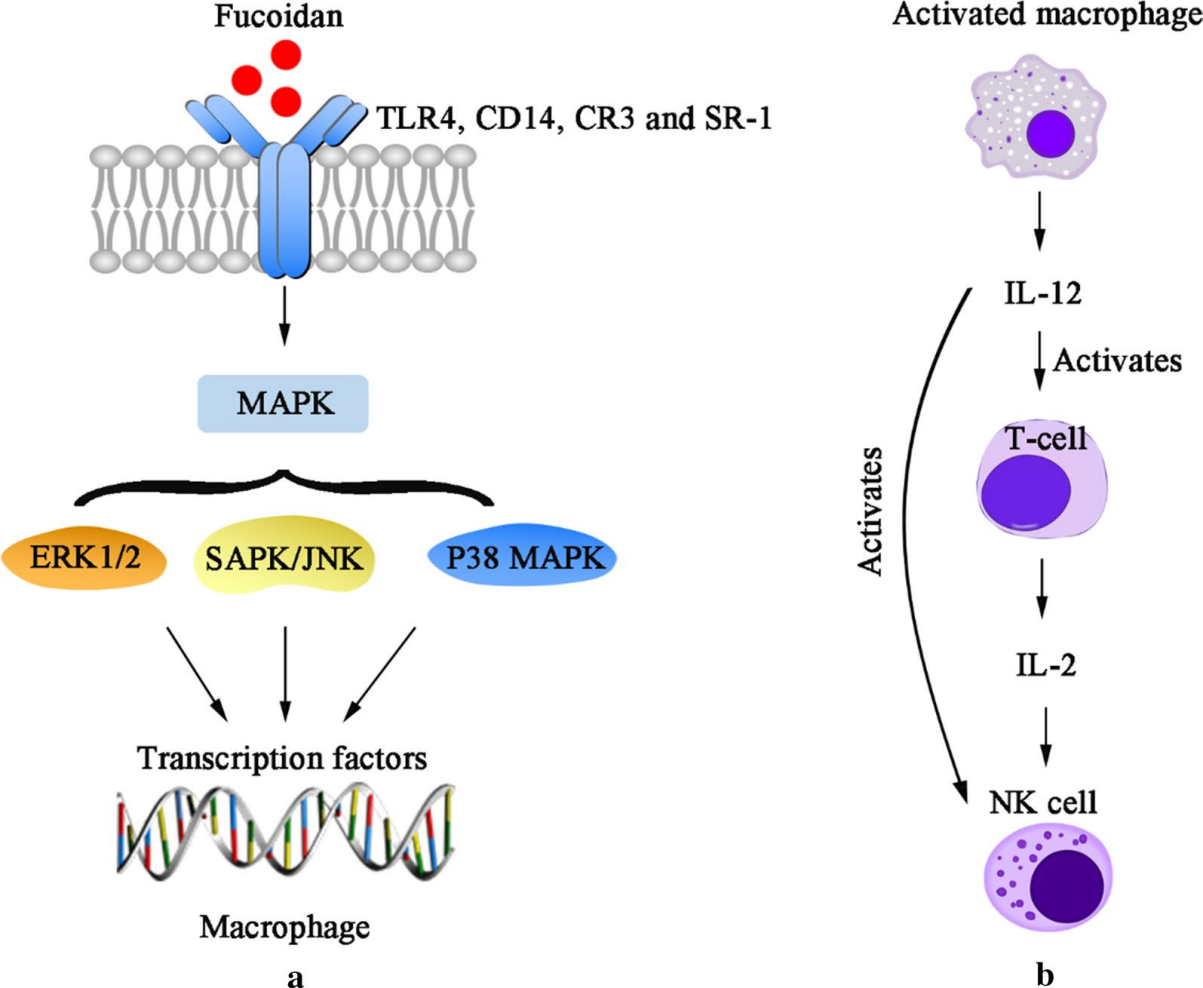
Action mechanism of fucoidan on activation of macrophages and NK cells [54]. **a** Fucoidan binds to specific glycoprotein receptors in macrophage cell membranes and activates MAPKs, thereby inducing the activation of transcription factors. **b** Activated macrophages release cytokines such as IL-12, which can activate T-cell and NK cell

Previous studies found that the anti-cancer mechanism of fucoidan mainly includes the following four aspects. First, fucoidan can suppress cancer cells’ proliferation by inhibiting the normal mitosis of them and regulating the cell cycle. Alekseyenko et al. injected fucoidan into C57 mice with transplanted Lewis lung adenocarcinoma. They discovered that tumor mass and the number of lung metastases were significantly lower than those without FUC, indicating that fucoidan effectively inhibited the metastasis and growth of the tumor cells in vivo [24]. Second, fucoidan can activate the apoptosis signals of cancer cells, induce apoptosis of them through related pathways, and thus produce an anti-cancer effect. Eun et al. co-cultured HT-29 and HCT116, human colon cancer cells, with fucoidan extracted from *Fucus vesiculosus*. From the results of apoptosis detection, fucoidan induced activation of caspase-3, -7, -8, -9, chromatin condensation and cleavage of poly(ADP-ribose) polymerase (PARP). These data indicates that fucoidan can induce HT-29 and HVT116 cells apoptosis through caspase-8 and -9 dependent pathways [49]. Third, fucoidan can inhibit the formation of VEGF, thereby suppressing the angiogenesis, cutting off the nutrient and oxygen supply of tumor, reducing the volume of it and blocking the spread and transfer of cancer cells. Tse-Hung et al. administered the fucoidan to mice implanted with Lewis lung cancer cells, and the levels of VEGF in serum and lung tissue were significantly reduced compared with those without FUC [50]. Koyanagi et al. found that whether natural or persulfated fucoidan can inhibit the mitosis and chemotaxis of VEGF165 in human umbilical vein endothelial cells by inhibiting VEGF165 to its cell surface receptors [51]. Fucoidan also inhibits neovascularization induced by human prostate cancer cells (DU-145) in mice [52]. Inhibition was also observed in mice with transplanted B16 melanoma [51]. These results show that the fucoidan’s anti-tumor activity is associated with its anti-angiogenic effect. Fourth, fucoidan can also activate immune system of the body, then enhancing the ability of natural killer cells and T cells to kill tumor cells. Farzaneh et al. fed the mice that have been transplanted with acute promyelocytic leukemia cells NB4 with fucoidan, and it was found that fucoidan could effectively increase the killing activity of NK cells (Fig. 2) [53].

### The research progress of fucoidan in vitro and in vivo

#### The anti-colon tumor effect of fucoidan

Colon cancer is one of the cancers in the world, which is very common [55, 56]. Vishchuk et al. applied fucoidan extracted from brown algae *Saccharina cichorioides* to the human colon cancer DLD-1 and found that it can inhibit tumor cell proliferation by suppressing the activity of epidermal growth factor [57]. Thinh et al. applied fucoidan extracted from *Sargassum mcclurei* to colon cancer DLD-1 cells. The results showed that fucoidan can inhibit cancer cells’ proliferation effectively with less cytotoxicity [58]. Kim et al. demonstrated that fucoidan induces HT-29 cell death and it may be owning to the downregulation of IGF-IR that signals through the IRS-1/PI3K/AKT pathway [59]. Wilfred et al. used fucoidan that was abstracted from *Undaria pinnatifida* to treat WiDr and LoVo human colon adenocarcinoma cell lines, then it was found that fucoidan can inhibit tumor cell proliferation effectively and the cytotoxicity to normal tissue cells is low [23]. Kim et al. studied fucoidan’s effects on apoptosis of HT-29 and HCT116. They found that the apoptosis of colon cancer cells induced by fucoidan is regulated by both the death mitochondria-mediated and receptor-mediated apoptotic pathways [49].

In vivo, Azuma et al. administered low, medium and high molecular weight fucoidan to colon 26 tumor-bearing mice and found that consumption of medium-molecular-weight fucoidan can inhibit the tumor growth significantly. They also illustrated that the survival time of mice in the low molecular weight or high molecular weight fucoidan group was substantially longer than that in the control group, and the number of NK cells in mice’s spleen was also significantly increased [26] (Table 1).

**Table 1:** Effect of fucoidan on colon cancer cells *in vitro*

Cell Type	Fucoidan source	Dose (μg/mL)	Effects on cell cycle	Effects on apopotosis pathways	Action characteristic	Action mechanism	Ref
DLD-1	*Saccharina*	50	–	–	Inhibit the binding of EGF receptor with EGF	Inhibit cell proliferation	[47]
DLD-1	*Sargassum*	100	–	–	Less cytotoxic colony formation inhibition	Inhibit cell proliferation	[48]
HT-29 HCT-116	*Fucus vesiculosus*	20	–	Caspase-8, 9, 7, 3 activation PARP, Bak, Bid, Fas ↑ Mcl-1, survivin, XIAP↓	–	Induce cell apoptosis	[40]
WiDr LoVo	*Undaria pinnatifida*	200–1000	–	–	Less cytotoxic	Inhibit cell proliferation	[18]
HT-29	*Fucus vesiculosus*	0–1000	–	IRS-1/PI3K/AKT pathway-related proteins↓ Ras/Raf/ERK pathway-related proteins ↓	–	Inhibit cell proliferation Induce cell apoptosis	[49]

*EGF* epidermal growth factor, *PARP* poly(ADP-ribose) polymerase, *XIAP* X-linked inhibitor of apoptosis protein

#### The anti-breast cancer effect of fucoidan

Yamasakimiyamoto et al. studied the apoptosis inducing impact of fucoidan on MCF-7 cells. They found that fucoidan induced chromatin condensation and fragmentation of nuclear interstitial DNA, etc. Researches have suggested that fucoidan can induce MCF-7 cells’ apoptosis through a caspase-8-dependent pathway [60]. Vishchuk et al. examined fucoidan’s impacts on breast cancer T-47D cell line, and learnt that fucoidan can inhibit T-47D cells’ proliferation effectively and had very low toxicity to mouse epidermal cells [57]. Wilfred et al. treated MCF-7 cells with fucoidan from *Undaria pinnatifida in* New Zealand and the fucoidan had been found to suppress tumor cell proliferation significantly and has extremely low cytotoxicity to normal tissue cells [23]. In addition, the scientists used 3-(4,5)-dimethylthiahiazo(-z-y1)-3,5-di-phenytetrazoliumromide (MTT) method to confirme that fucoidan could decrease the number of viable cells. The MCF-7 cells were detected by flow cytometry. It was found that G1 arrest is associated with a decrease in gene expression. This study’s overall results indicated that fucoidan can induce apoptosis and G1 phase arrest by regulating apoptosis-related gene expression and cell cycle [61]. Fucoidan can reverse the EMT effectively, which was induced by TGFβ receptors (TGFRs). It can also up-regulate epithelial markers, down-regulate interstitial markers and decrease the expression of transcriptional repressors Snail, Slug and Twist, thereby inhibiting the growth of MDA-MB-231 cells and reducing the formation of its cell colonies. An in vivo experiment by the same group involving administrating fucoidan to 4T1-xenografted mice shown that in comparison with control group that were injected with PBS solution, the tumor volume was significantly reduced, and the average number of metastatic tumor nodules in lungs was also significantly reduced. This research proved that fucoidan can prevent the proliferation and metastasis of 4T1 cells effectively [27] (Table 2).

**Table 2:** Effect of fucoidan on breast cancer cells *in vitro*

Cell type	Fucoidan source	Dose (μg/mL)	Effects on cell cycle	Effects on apopotosis pathways	Action characteristic	Action mechanism	Ref
MCF-7	*Cladosiphon*	1000	Sub-G1 fraction↑	PARP cleavage Caspase-7,8,9 ↑ Cytochrome C, Bax, Bid↑	–	Induce cell apoptosis	[50]
T-47D	*Saccharina*	50	–	–	Less cytotoxic inhibit the binding of EGFReceptor with EGF	Inhibit cell proliferation	[47]
MCF-7	*Fucus vesiculosus*	300	G1 phase arrest Sub-G1 fraction↑ Cyclin D1, CDK-4 gene expression↓	Caspase-8 activation Cytochrome C, Bax ↑ Bcl-2↓ Release of APAf-1↑	ROS↑	Induce cell apoptosis	[51]
MDA-MB-231	*Fucus vesiculosus*	90–120	–	The protein expressionof phosphorylated Smad2/3, Smad4↓	–	Inhibit cell proliferation	[22]
MCF-7	*Undaria pinnatifida*	2004–1000	–	–	–	Inhibit cell proliferation	[18]

*PARP* poly(ADP-ribose) polymerase, *EGF* epidermal growth factor, *ROS* reactive oxygen species

#### The anti-lung cancer effect of fucoidan

Dimitri et al. treated human non-small-cell bronchopulmonary carcinoma line (NSCLC-N6) with fucoidan extracted from *Bifurcaria bifurcata* on the Atlantic coast, and found that tumor cells were irreversibly inhibited [62]. Wilfred et al. treated human lung cancer A549 cells with fucoidan and found that it could inhibit tumor cells’ proliferation significantly and had low cytotoxicity to normal tissue cells [23]. Its relative mechanism of action has been elucidated in similar experiments. Hye-Jin et al. also treated A549 cells with fucoidan extracted from *Undaria pinnatifida*. In addition to its strong anti-proliferative activity, it was also found that fucoidan could down regulate p38 mitogen-activated protein kinase (p38 MAPK) and phosphatidylinositol 3-kinase/protein kinase B (PI3K/Akt), and the pathway to induce A549 cells apoptosis [63]. Madhavarani et al. demonstrated that fucoidan purified from *Turbinaria conoides* induces reduction in survival rate of A549 cells in a dose-dependent way. They also found that it was not cytotoxic to a non-tumorigenic human keratinocyte cell line of skin tissue (HaCaT) [55]. Huang et al. cultured the Vero normal kidney epithelial cells and Lewis lung carcinoma cells in different concentrations of fucoidan solution. MTS assay showed that the LLC cells growth was significantly prevented in a dose-dependent way, but not in normal kidney cells.

An in vivo experiments indicated that fucoidan could alleviate the viral symptoms of C57BL/6 mice and inhibit the lung metastasis of mice with transplanted Lewis lung cancer [50]. In another research, Alekseyenko et al. also used C57BL/6 mice inoculated with Lewis lung cancer cells to explore the combined effect of cyclophosphamide and fucoidan as an adjuvant which showed that the repeated injection of fucoidan enhanced the cyclophosphamide’s anti-metastatic effect, but did not enhance its anti-tumor effect. Cyclophosphamide’s toxic effect is enhanced by a single injection of a 25 mg/kg of fucoidan [24]. Hsien-Yeh et al. researched the impact of fucoidan in sequential therapy (Cisplatin-based). They illustrated that fucoidan induce apoptotic responses by upregulating the expression of cleaved caspase-3 and poly (ADP ribose) polymerase (PARP). The research in LLC-1 cells transplanted C57 mice revealed that the combination of cisplatin and fucoidan was more effectual at repressing tumor volume compared with using them alone [22]. The relevant studies have found that fucoidan can suppress the new blood vessels that is induced by Sarcoma 180 cells in mice [51]. The experiment demonstrated that fucoidan can exert an effective anti-tumor effect through its anti-angiogenic ability [24] (Table 3).

**Table 3:** Effect of fucoidan on lung cancer cells *in vitro*

Cell type	Fucoidan source	Dose (μg/mL)	Effects on cell cycle	Effects on apopotosis pathways	Action characteristic	Action mechanism	Ref
A549	*Undaria pinnatifida*	10–200	sub-G1 fraction↑	Bcl-2, p38, Phospho-PI3K/Akt, procaspase-3↓ Bax, caspase-9, Phospho-ERK1/2 ↑ PARP cleavage	NK-cell ↑	Inhibit cell proliferation Induce cell apoptosis	[53]
NSCLC-N6	*Bifurcaria bifurcata*	2–9	G1 phase arrest	–	The growth arrest is irreversible	Inhibit cell proliferation	[52]
Lewis lung carcinoma cells	*Fucus vesiculosus*	50–400	–	NF-κB↓	Inhibit VEGF,MMPs	Inhibit metastasis	[41]
A549	*Undaria pinnatifida*	200–1000	–	–	Less cytotoxic	Inhibit cell proliferation	[18]
A549 H1975	*Fucus vesiculosus*	0–400	–	Caspase-3↑ PARP cleavage	TLR-4 mediated	Inhibit cell proliferation Induce cell apoptosis	[17]
A549	*Turbinaria conoides*	10–1000	G0/G1 phase arrest	**–**	–	Inhibit cell proliferation Induce cell apoptosis	[45]

*PARP* poly(ADP-ribose) polymerase, *VEGF* vascular endothelial growth factor, *MMPs* matrix metalloproteinases

#### The anti-hepatoma effect of fucoidan

**Fig. 3:**
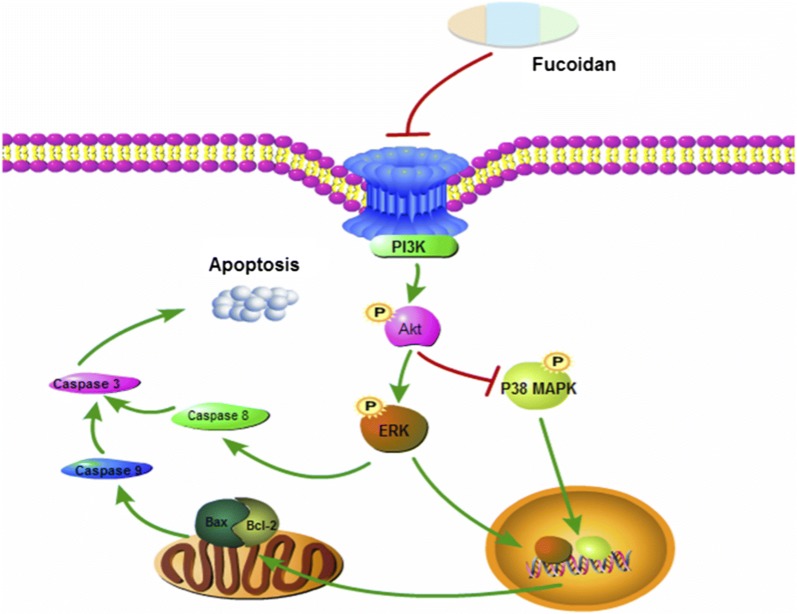
The molecular mechanism of fucoidan’s anti-tumor activity [65]

Fucoidan also expresses anti-tumor activity by inhibiting cell cycle and inducing cancer cells apoptosis. After treatment of human hepatoma SMMC-7721 cells with fucoidan, it showed significant growth inhibition and apoptosis. There are several typical features such as mitochondrial swelling, vacuolization, chromatin condensation or marginalization and decreased number. The study also found that fucoidan-induced SMMC-7721 cells apoptosis was associated with decreased consumption of glutathione (GSH). This process also increased the level of ROS in cells, with the damage of the ultrastructure of the mitochondria and depolarizing the mitochondrial membrane potential. These evidences suggest that fucoidan can induce human hepatocellular carcinoma SMMC-7721 cells apoptosis via ROS-mediated mitochondrial pathway [64]. In another experiment, scientists researched the effects of fucoidan on microRNA expression and found that it significantly upregulated the microRNA-29b(miR-29b) in human HCC cells. The induction of miR-29b was in a dose-dependent relationship with the inhibition of its downstream target DNA methyltransferase 3B (DNMT3B). The messenger RNA and the protein levels of tumor metastasis suppressor gene 1 (MTSS1), which was inhibited by DNMT3B, were significantly raised after remedy with fucoidan. In addition, fucoidan also down-regulated the transforming growth factor (TGF) receptor and SMAD signal in hepatoma cells. These effects could inhibit the degradation of extracellular matrices and reduce the invasive activity of HCC cells [35]. The BEL-7402 and LM3 cell lines are treated by fucoidan and the result indicated that the role of fucoidan in inhibiting cell proliferation is mediated through the p38MAPK/ERK pathways. Fucoidan inhibits the activation of PI3K, which leads to the inhibition of ERK and the activation of MAPK. The ratio of Bcl-2 to Bax decreased, resulting in mitochondrial dysfunction. Then the caspase release increased, causing apoptosis (Fig. 3) [65].

Tumor metastasis is one of the important causes of cancer patients’ death. Blood and lymphatic metastasis are the main ways for cancer cells to form distant metastases. This is a complicated biological process with multiple genes. The process of metastasis is also related to biological activities of cancer cells, in the terms of growth, invasion, blood circulation, lymphatic metastasis, etc. Cho et al. found out that the anti-metastasis effect of fucoidan and the role of key signals in regulating metastasis. Both experiments have proved that it can stop the invasiveness of liver cancer cells by inhibiting the N-myc downstream regulated gene 1(NDRG-1)-dependent factor ID-1 [66]. In addition, fucoidan inhibited the invasion of hepatocarcinoma cells by up-regulating NDRG-1/CAP43, which was mediated by extra-cellular signal-regulated kinases 2/1 (p42/44 mapk). It was also elucidated that fucoidan reduces the metastasis of hepatoma cells in vivo by up regulating the expression of p42/44 mapk-mediated vacuolar membrane protein 1(1VMP-1) under normoxia, and it also reduces the apoptosis of hepatocytes induced by bile acid through the inhibition of caspase-8, caspase-7 and the activation of Fas related death domain. In order to study whether fucoidan has anti-metastasis activity in the liver metastasis model of MH134 cells, Yuri et al. found that the number of hepatic metastasis focus was largely lower than that of the control group, and the sum of the maximum diameter of liver metastases in fucoidan treated mice was lower than that of the control group [25] (Table 4).

**Table 4:** Effect of fucoidan on hepatoma carcinoma cells *in vitro*

Cell type	Fucoidan source	Dose (μg/mL)	Effects on cell cycle	Effects on apopotosis pathways	Action characteristic	Action mechanism	Ref
Huh6 Huh7 SK-Hep1 HepG2	*Sargassum*	200	–	TGF-β R1, 2↓ Phospho-Smad2/3↓ Smad 4 protein↓	Colony formation inhibition	Inhibit cell proliferation	[30]
SMMC-7721	*Undaria pinnatifida*	65.2–1000	Accumulate in the S-phase	Livin, XIAP mRNA ↓ Caspase-3, -8, -9 ↑ Bax-to-Bcl-2 ratio↑ Cytochrome C ↑	The quantity of mitochondria ↓ ROS ↑ Depolarization of the MMP	Inhibit cell proliferation Induce cell apoptosis	[54]
Huh-7 SNU-761 SNU-3085	*Fucus vesiculosus*	1000	–	Caspase-7, -8, -9 ↑	–	Inhibit cell proliferation	[55]
Huh-BAT Huh-7 SNU-761	*Fucus vesiculosus*	100, 250, 500,1000	sub-G1 fraction↑	Bax, Bid, Fas↑ Caspase-7, -8, -9 cleavage Phosphorylated-p42/44↑	–	Inhibit cell proliferation Inhibit metastasis Induce cell apoptosis	[20]

*IAP* inhibitor *of apoptotic protein, ROS* reactive oxygen species*, MMP* mitochondrial membrane potential

#### The anti-leukemia effect of fucoidan

Several researches on anti-leukemia effect of fucoidan achieve good results. Jin et al. studied the signaling pathway of fucoidan-mediated apoptosis. Fucoidan treatment of HL-60 cells could induce activation of caspases-3, -8, -9, and change of the mitochondrial membrane permeability [67]. The same research results are reflected in other experiments. Hyun et al. found that the increase in apoptosis is related to the caspases hydrolase, the cleavage of Bid, insertion of the Bax into mitochondria before apoptosis, the release of the cytochrome c from mitochondria to cytoplasm and the loss of mitochondrial membrane potential in U937 cells. They also found that caspase inhibitors inhibited apoptosis induced by fucoidan, indicating that apoptosis depended on caspase activation. In addition, fucoidan can effectively activate the p38 mitogen-activated protein kinase (MAPK) and p38 MAPK inhibitors, and largely went against fucoidan-induced apoptosis by inhibiting Bax translocation and caspases activity, suggesting that the activation of p38 MAPK may play an essential part in fucoidan-induced apoptosis. Hyun et al. also found that fucoidan significantly attenuated the overexpressing of Bcl-2 in U937 cells [68]. Therefore, they tried to ascribe some of the biological functions of p38 MAPK and Bcl-2 to their capability to suppress fucoidan-induced apoptosis. Farzaneh et al. explored the cytotoxicity and anti-tumor activity of fucoidan on human acute myeloid leukemia cells. The results revealed that fucoidan inhibited the proliferation and induced apoptosis of NB4 and HL60 by endogenous and exogenous pathways. In NB4 cells, apoptosis was affected by caspase, while pretreatment with pan-caspase inhibitors can significantly attenuate apoptosis. The significant up-regulation of P21, WAF1 and CIP1 resulted in cell cycle arrest. Based on the study of fucoidan on NB4 transplanted mice, researchers focused on tumor size, cytotoxic activity and NK cells, then they found that fucoidan can significantly delay the xenograft tumor growth and increase the cytolytic activity of NK cells. These results showed that fucoidan could be a useful drug to treat some types of leukemia [53].

Yang et al. studied the antitumor activity of fucoidan in diffuse large B cell lymphoma (DL-BCL) cells in vivo and in vitro. The findings showed that fucoidan caused G0/G1 cell cycle arrest and it also caused the loss of MMP in lymphoma cells, and the cytochrome c and apoptosis-inducing factors released from the mitochondria into the cytoplasm, then induced apoptosis of lymphoma cells [69]. Scientists studied the fucoidan on tumor growth of mouce A20 leukemia cells, and they also researched the effects on T cell-mediated immunity response in T cell receptor transgenic (DO-11-10-Tg) mice. In mice that added fucoidan to food, the lytic activity of ovalbumin that inhibited lymphoma cell transfection was enhanced, and the killing effect of NK cells was also significantly enhanced [70] (Table 5).

**Table 5:** Effect of fucoidan on leukemia cells i*n vitro*

Cell type	Fucoidan source	Dose (μg/mL)	Effects on cell cycle	Effects on apopotosis pathways	Action characteristic	Action mechanism	Ref
HL-60 NB4 THP-1	*Fucus vesiculosus*	150	Sub-G1 fraction ↑	PARP cleavage Caspase-8, 9, 3 ↑ Mcl-1, Bid ↓	ERK1/2, MEK1/2, JNK ↑	Induce cell apoptosis	[56]
SUDHL-4 OCI-LY8 NU-DUL-1 TMD8 U293 DB	*Fucus vesiculosus*	50, 100, 200	G0/G1 phase arrest CyclinD1, CDK4, CDK6↓ p21 ↑ E2F1 ↓	PARP cleavage Cleaved Caspase-8, 9, 3 ↑	–	Induce cell apoptosis	[58]
NB4 HL60	*Fucus vesiculosus*	12.5, 25, 50, 100	Sub-G0/G1 fraction ↑ p21, WAF1, CIP1 ↑	Caspase-3, 8, 9 ↑ PARP cleavage Bax ↑	Activation of ERK1/2, AKT ↓ NK cell ↑	Inhibit cell proliferation Induce cell apoptosis	[44]
U937	*Fucus vesiculosus*	20–100	Sub-G1 fraction ↑	Caspase-3, 8, 9 ↑ PARP cleavage Bax↑ Bid, Bcl-xl, MMP↓	p38MAPK activation	Inhibit cell proliferation Induce cell apoptosis	[57]

*PARP* poly(ADP-ribose) polymerase*, ER* extracellular signal-regulated kinase, *MEK:* MAPK kinase*, MAPK* mitogen-activated protein kinase, *JNK* Jun NH2-terminal kinase*, MMP* mitochondrial membrane potential

#### The anti-human bladder cancer effect of fucoidan

In 2014, Hye et al. first reported the impact of fucoidan on the growth of bladder cancer cells. The results found that fucoidan reduced the viability of T24 cells by inducing G1 cell cycle arrest. They also found that this arrest caused by fucoidan is related to the increased expression of the CDK inhibitor and the dephosphorylation of pRB. This study also found the loss of MMP and the release of cytochrome c from the mitochondria to cytoplasm. They confirmed the mitochondrial dysfunction and growing Bax/Bcl-2 expression ratio after treatment with fucoidan. The Apoptosis caused by fucoidan was also combined with the up-regulation of Fas, truncation of Bid, and sequential activation of caspase-8. In addition, fucoidan significantly increased the activation of caspase-9/3, decreased the degradation of PARP and the expression of IAPs. These observations indicated that fucoidan is a significant mediator of the interaction between the caspase-dependent endogenous and exogenous apoptotic pathways in T24 cells [40]. The scientists treated human bladder cancer cells 5637 with fucoidan and it was found that fucoidan suppressed tumor growth, which is manifested in promoting the expression of cyclin-dependent kinase inhibitor 1 (p21WAF1) and inhibiting the expression of cyclin and cyclin-dependent kinases. It had also been found that treatment with fucoidan can inhibit metastasis and infection of bladder cancer cells. The similar results were also found in T24 cells [71]. Han et al. reported that fucoidan-induced human bladder cancer 5637 cells apoptosis was linked with the increasing in the ratio of Bax/Bcl-2, structural destruction of mitochondrial membranes, and the releasing of cytochrome C. Under the same experimental conditions, scientists found that fucoidan reduces the expression of human telomerase reverse transcriptase (hTERT), proto-oncogene transcription factor (c-myc) and stimulating protein 1(Sp1). They also discovered that fucoidan enhanced the apoptosis and decreased telomerase activity by inhibiting the activation of the PI3K/Akt signaling pathway. The experimental data indicated that fucoidan-induced apoptosis and inhibition of telomerase activity are mediated by the inactivation of PI3K/Akt pathway dependent on reactive oxygen species [72].

Meng-Chuan et al. found that low-molecular-weight fucoidan (LMWF) can inhibit the formation of hypoxia-stimulated H_2_O_2_, accumulation of hypoxia-inducible factor-1, secretion of transcriptionally active vascular endothelial growth factor, and the migration and invasion of hypoxic human bladder cancer cell T24. It also inhibited the hypoxia-activated phosphorylation of PI3K/AKT/mTOR/p70S6K/4EBP-1 signaling in T24 cells [73].

#### The anti-tumor potential in other types of cancers

Vishchuk et al. treated melanoma RPMI-7951 cell line with fucoidan and found that fucoidan could regulate the tumor cell cycle and affect the tumor cell mitosis [57]. Oral intake of fucoidan (5 mg/kg) was effective for suppressing tumor growth on melanoma B16 cell transplanted mice. It was obtained that fucoidan could suppress the expression of VEGF and inhibit tumor angiogenesis, and the oversulfated fucoidan seems more effective [51]. Boo et al. once cultured PC-3, human prostate cancer cell, with fucoidan extracted from *Undaria pinnatifida.* The dose is 200 μg/mL. They found that fucoidan activated ERK1/2 MAPK, inhibited p38 MAPK and PI3K/AKt signaling pathways and then promoted apoptosis of PC-3 [74]. Gang-Sik et al. fed human prostate cancer DU-145 cells transplanted mice with fucoidan and found that p38 MAPK and PI3K/Akt signaling pathways were inhibited by fucoidan, while apoptosis was enhanced. The gene expression of Bcl-2 was inhibited and caspases-9 was activated, triggering DNA damage [6]. The therapeutic effect of fucoidan on DU-145 cells was studied by Xin et al. In vitro, the researchers treated DU-145 with fucoidan with a dose of 100–1000 μg/mL. They discovered that fucoidan went against the proliferation and activity of DU-145 cells and against the migration and management of cells in matrix. In vivo, they injected mice with DU-145 cells to establish xenotransplantation models. The oral gavage for 28 days with 20 mg/kg of fucoidan significantly inhibited the growth of tumors and angiogenesis, decreased hemoglobin content in tumor tissues, and decreased mRNA expression of CD31 and CD105. In addition, the phosphorylated JAK, STAT3 and the activation of VEGF, Bcl-xL and Cyclin D1 were decreased significantly after fucoidan treatment. The above results indicated that the anti-tumor and anti-angiogenic effects of fucoidan may be mediated via the JAKSTAT3 pathway [52]. Hyun et al. explored the possible mechanism of fucoidan on the anti-proliferative effect of human gastric adenocarcinoma AGS cells in vitro. The results indicated that fucoidan has the ability to down-regulated the expression of Bcl-2 and Bcl-xL, decreased the MMP, and cleavaged of the poly-(ADP-ribose) polymerase protein. These data suggested that fucoidan can inhibit AGS cells’ growth effectively by inducing autophagy and apoptosis [75]. Scientists studied the effects of fucoidan imposed on the uterine sarcomas cells ESS-1 and MES-SA, and carcinosarcoma cell lines SK-UT-1 and SK-UT-1B, and its toxic effect on the fibroblasts of human skin. The results indicated that fucoidan significantly reduced the viability of SK-UT-1, SK-UT-1B and ESS1 cell lines, while the dosage of fucoidan in their study had no significant effect on normal cell proliferation. In addition to MES-SA, all tested cells were affected by fucoidan, which increased the percentage of cells in the G0, sub-G1 or G1 phase. They found that fucoidan not only affects cell proliferation, but also selectively induces apoptosis of uterine sarcomas and carcinosarcoma cells, which has potential cytotoxicity [76].

## Clinical research

In recent years, there are few studies on the potential systemic effects of oral fucoidan at home and abroad, and most of them are carried out in vitro or in mice. There are few clinical studies mainly due to the following reasons: The molecular structure of fucoidan is complex and diverse, it is difficult to ensure the accuracy and representativeness of the study. In addition, the absorption of fucoidan after oral administration is small, and the concentration of fucoidan within the body cannot be accurately measured [30]. Fucoidan has not yet been certified as a drug, so large-scale clinical trials cannot be conducted [77]. With the development of a large number of anti-tumor effects and related mechanisms of fucoidan, scientists have found that the low toxicity and anti-inflammatory properties of fucoidan make it an adjuvant therapy for tumor patients based on conventional treatment [78]. Stephen et al. underwent a 12-week, double-blind, controlled experiment at random on patients with osteoarthritis. The efficacy of treatment was measured by comprehensive osteoarthritis test, and the safety was measured by evaluating liver function, cholesterol, hematopoietic function, renal function and closely monitoring of adverse events. The result showed that the 300 mg intake of fucoidan is safe and well tolerated in humans. However, fucoidan has no significant effect in relieving OA symptoms compared with placebo [9]. In a clinical study in Japan, the researchers selected 13 patients with HTLV-1 associated myelopathy/tropical spastic paralysis (HAM/TSP) for enrollment. The patient took 6 g of fucoidan orally daily and continued to take it for at least 6 months. The relevant results showed that compared with the control group, the previral DNA load of patients who took fucoidan significantly decrease by about 42.4% [39]. The first time, Hidenori et al. provided evidence for the anti-inflammatory effects of fucoidan on advanced cancer patients. The researchers conducted a prospective open-label clinical study that included 20 patients with advanced cancer. The patient took oral fucoidan 4 g daily for at least 4 weeks. The results of the experiment showed that major pro-inflammatory cytokines, including interleukin-1β (IL-1β), IL-6 and tumor necrosis factor-α (TNF-α), showed a significant decrease after 2 weeks of continuous ingestion of fucoidan. But the quality of life scores, including fatigue, did not change significantly during the study period [79]. Shreya et al. investigeted the effects of fucoidan extracted from *Undaria pinnatifida* on the pharmacokinetics of two common used hormone therapies, letrozole and tamoxifen, in breast cancer patients. The enrolled patients received 1 g of fucoidan daily for 3 weeks. The results showed that the steady-state plasma concentrations of letrozole, tamoxifen and tamoxifen metabolites did not change significantly after binding with fucoidan. However, there wasn’t any significant differences in toxicity were observed during the period. These results indicated that the use form and dose of fucoidan can be used simultaneously with letrozole and tamoxifen without significant risk of interaction [80]. Low-molecular-weight fucoidan (LMWF) is a food supplement which is widely used in cancer patients. Hsiang et al. tested the efficacy of LMF as a complementary therapy for chemotherapy drugs and target drugs in patients with metastatic colorectal cancer. They underwent a prospective, randomized, double-blind, controlled trial of up to 6 months with a total of 54 patients. In the experimental group, 28 cases took 4 g of fucoidan everyday, and in the control group, 26 cases took 4 g of cellulose everyday. According to the result, there was a significant difference in disease control rate (DCR) between the experimental group and the control one, 92.8% and 69.2% respectively. To the best of our knowledge, this is the first clinical trial to evaluate the efficacy of LMWF as a complementary treatment in metastatic colorectal cancer (mCRC) patients. The results demonstrated that LMWF combined with chemotherapy targeting drugs can largely improve the DCR [81].

## Adverse effects of fucoidan

As of now, there are few studies on the side effects of fucoidan. An in vivo experiment using SD rats in South Korea tested the toxicity of oral fucoidan. Rats took fucoidan 150–1350 mg/Kg daily for 28 days. The experimental results showed that there were no obvious abnormalities in the vital signs of rats and only the serum urea nitrogen of female showed an increase. In addition, rats taking 1350 mg/Kg fucoidan showed a reduction in relative liver weight. Generally speaking, these findings suggested that fucoidan has no evident toxic effects under this feeding pattern [82]. Chung et al. demonstrated the potential toxic effects of fucoidan in vitro and in vivo. In the Ames tests, fucoidan at a concentration of 500 μl per plate did not show a significant effect of inducing colony reproduction. However, the thyroid weight of rats increased significantly after taking 2000 mg/Kg of fucoidan daily. The ALT and lipid metabolism test results of rats also showed significant changes. The above results suggest that fucoidan may have potential liver toxicity [37]. In a clinical study, 4 of 17 patients who took 6 g of fucoidan daily showed symptoms of diarrhea, and it could be significantly relieved after stopping the fucoidan [39]. However, due to the lack of relevant research, it is not yet possible to accurately assess the adverse effects of fucoidan.

## Conclusions

At present, scientists have demonstrated the anti-tumor effect of fucoidan, including inhibiting the growth, metastasis, angiogenesis and inducting apoptosis of various cells of tumor in vitro and in vivo [19, 40–42]. Furthermore, fucoidan, as an immunmodulatory molecule, reduces side effect when administrating with chemotherapy drugs and radiotherapy [44]. In summary, fucoidan has great potential in cancer treatments. However, due to the lack of research on the potential pharmacokinetic interactions between fucoidan and traditional tumor drugs, there are few clinical data about fucoidan. In the future, more research will be conducted to explore its mechanisms and functions in the treatment of cancer. More large-scale and multi-center blind-controlled trials are needed to determine the efficacy of fucoidan support for cancer patients, especially in chemotherapy patients. In the future, fucoidan may become a favorable and natural anticancer therapeutic or auxiliary drug, opening a new direction for new anticancer drugs’ evolution.

## Data Availability

All data generated or analysed during this study are included in this published article and its supplementary information files.

## Abbreviations

FUC: Fucoidan
PARP: Poly(ADP-ribose) polymerase
VEGF: Vascular endothelial growth factor
VEGF165: Vascular endothelial growth factor 165
PI: Propidium iodide
EMT: Epithelial to mesenchymal transition
TGFβ: Transforming growth factor β
TGFRs: Transforming growth factor β (TGFβ) receptors
ROS: Reactive oxygen species
miR-29b: MicroRNA-29b
TGF: Transforming growth factor
NDRG-1: N-myc downstream regulated gene 1
CAP43: Calciumasso-ciated protein 43
VMP-1: Vacuolar membrane protein 1
MMP: Mitochondrial membrane potential
MAPK: Mitogen-activated protein kinase
APL: Acute promyelocytic leukemia
AP-1: Activator protein-1
hTERT: Human telomerase reverse transcriptase
Sp1: Stimulating protein 1
LMWF: Low-molecular-weight fucoidan
DCR: Disease control rate
